# Enrollment and retention of female sex workers in HIV care in health facilities in Mbarara city

**DOI:** 10.3389/frph.2022.1089663

**Published:** 2023-01-18

**Authors:** Bridget Arinaitwe, Philbert Ariho, Christine Hilda Naturinda, Brian Byoleko, Anitah Base, Humphrey Atwijukiire, Hariat Matavu, Jane Kabami, Celestine Obua, Edith K. Wakida, Anne Otwine

**Affiliations:** ^1^ Department of Nursing Science, Bishop Stuart University, Mbarara, Uganda; ^2^ Department of Public Health and Biomedical Sciences, Bishop Stuart University, Mbarara, Uganda; ^3^ Department of Nursing, Kabale University, Kabale, Uganda; ^4^ Department of Medicine, Mbarara University of Science and Technology, Mbarara, Uganda

**Keywords:** HIV healthcare, female sex workers, enrollment, retention, Mbarara City

## Abstract

**Background:**

Sex work is a global driver of the HIV epidemic, and the risk of acquiring HIV is 13 times higher for female sex workers (FSWs) compared to the general population. The enrollment and retention of FSWs in HIV care is a challenge and has been a major contributing factor to increased new HIV infections.

**Methods:**

We conducted a qualitative study among 30 FSWs and 21 healthcare workers (HCWs) working in antiretroviral therapy (ART) clinics at the selected three primary health facilities in Mbarara City, Southwestern Uganda. The study participants were enrolled by both purposive and snowball sampling techniques. We obtained informed consent from all the participants, and data were collected using in-depth interviews and thematically analyzed.

**Results:**

Three themes emerged as facilitators toward enrollment and retention of FSWs into HIV care, namely, (1) good health living, (2) receptive HCWs and availability of health services, and (3) community outreach services and peer support. The barriers are summarized into four major themes: (1) stigma, community discrimination, and beliefs; (2) social obstacles; (3) adverse effects of ART; and (4) inadequate services at the health facilities.

**Conclusion:**

FSWs are challenged by unsupportive environments and communities where they live and work, which hinders their enrollment and retention in HIV care. Creating awareness of the utilization of HIV care services and extending such services to hotspot communities could enhance the response of HIV-positive FSWs to ART.

## 1. Introduction

Sex work is a global driver of the HIV epidemic, with approximately 11.5%–18.6% of new HIV infections in women involved in sex work (1). Globally, the risk of acquiring HIV is 13 times higher for female sex workers (FSWs) compared to the general population (2). In sub-Saharan Africa, more than half of all new HIV infections are attributed to women engaged in sex work and their sexual partners (3); HIV prevalence among FSWs is high, estimated at 50% in Rwanda (4), 37% in Uganda (5), and 26.6% in Tanzania (6), compared to 6.5% among the general population of adult women (7).

FSWs are defined as women receiving money or gifts in exchange for consensual sexual services or erotic performances, either regularly or occasionally, and because of having multiple partners and sexual contacts, they are vulnerable to sexual health risks (8). FSWs living with HIV in sub-Saharan Africa have a poor engagement in HIV care and treatment (9); their enrollment and retention in HIV care is a challenge and has been a major contributing factor to increased new HIV infections (10). Retention in HIV care is defined as the continued engagement in health services, from enrollment in care to discharge or death of an individual living with HIV (11).

Sex workers are often marginalized, criminalized, and stigmatized by the societies in which they live, which increases their vulnerability to HIV infection (12). In Uganda, sex work is illegal, which negatively affects HIV care-seeking behaviors among FSWs (13) due to fear of stigma, discrimination, criminalization, and confidentiality issues (14). Thus, FSWs are a socially hidden and highly mobile population, transitioning in and out of sex work as dictated by economic needs (15). FSWs require tailored public health interventions that address critical gaps in HIV prevention and testing, linkage, enrollment, and retention to antiretroviral therapy (ART) (15).

Several models of service delivery among sex workers (integrated facility-based and stand-alone targeted services as well as outreach services delivered at various hours through the day and night) have been described (16). In Uganda, the Ministry of Health (MOH) developed guidelines for service delivery among sex workers (17); however, it is unclear to what extent these services meet the needs of FSWs and what gaps still exist.

According to the Ministry of Health in Uganda, Mbarara City, in the southwestern region, ranks second in the country with the highest prevalence of HIV at 13.5% despite the availability of health facilities and HIV care services; this could be due to poor enrollment and retention of FSWs in HIV care, which increases the risks of spreading HIV among themselves, their partners, and the entire community (18). Therefore, this study explored the factors linked to the enrollment and retention of female sex workers in HIV care in Mbarara City.

### 1.1. Specific objectives

The specific objectives are to assess factors that contribute to the linkage of FSWs living with HIV in care at individual and community levels.

### 1.2. Research questions

What factors contribute to the linkage to HIV care for FSWs living with HIV in Mbarara City?

## 2. Materials and methods

### 2.1. Study design

This was a descriptive cross-sectional study that employed a phenomenological qualitative approach. We collected data by conducting in-depth interviews to explore the facilitators and barriers toward enrollment and retention of FSWs in HIV care in Mbarara City. Qualitative research is an approach that seeks to tell the story of a particular topic under study based on the experiences of the individuals and/or groups using their own words. We conducted this study between February and April 2022.

### 2.2. Study setting

We conducted this study in three public healthcare facilities in Mbarara City, Southwestern Uganda. Mbarara is the largest city in Southwestern Uganda, located 269.6 km from Kampala, the main capital city. The facilities offer free HIV counseling and testing services, ART adherence counseling, laboratory services, and prevention of mother-to-child transmission. Primary health facilities were purposively selected because they offer HIV care services to FSWs within the city.

### 2.3. Study participants

We recruited and enrolled (1) FSWs living with HIV aged 18 years and above, staying and or working in Mbarara City, that have ever enrolled for HIV care in the selected three public health facilities in Mbarara City; and (2) health care workers (HCWs) working in the ART clinics in the selected public health facilities within Mbarara City for the last 12 months. The selected HCWs have been in close contact and interacting with FSWs. The study excluded FSWs who were critically ill and HCWs who were not available at the time of data collection.

### 2.4. Sample size

We enrolled 30 FSWs and 21 HCWs in the study as determined by the saturation of information. Study participants aged 18 years or older, able to provide informed consent, were recruited in the study.

### 2.5. Sampling criteria and data collection

1) HCWs in ART clinics: We purposively recruited HCWs who had worked in the ART clinics for the last 12 months and have been in close contact and interacting with the FSWs. We thought 12 months is enough period for any HCW to have experienced the facilitators and barriers encountered by FSWs while enrolling and retaining in HIV care.
2) FSWs: With the help of expert clients and HCWs, we purposively identified FSWs who were retained in HIV care. Using the snowball sampling technique, we identified other FSWs who had ever enrolled in HIV care, whether retained on care or not.

We collected data using an interview guide and an audio recorder. We translated the interview guide developed from the existing literature into a local language (Runyankole) for the participants who did not understand English to freely give information. The study tools were pilot-tested at Kitagata Hospital and Mbarara Regional Referral Hospital. The final adjustments were made to make the questions clear to the participants.

We obtained written informed consent from the participants before they participated in the study. The interviews were conducted in private and quiet places, and each interview lasted between 30 and 45 min. For bracketing purposes, the researchers documented the participant's experiences and nonverbal cues.

### 2.6. Data management and analysis

The recorded interviews were transcribed verbatim immediately after data collection. The researchers listened to the recordings and compared them with the transcripts to ensure that the transcribed information was from the recordings. The transcripts were translated from Runyankole to English and backtranslated to Runyankole by independent translators to check for consistency.

Data were analyzed manually using inductive thematic content analysis. This was done through the following steps: reading and rereading the participants’ descriptions of the phenomenon to acquire a feeling for their experience and roles and making sense of their account. Thereafter, similar statements were coded. Similar codes were categorized to form subthemes. Finally, related subthemes were merged to form themes.

### 2.7. Ethical consideration

This study was conducted according to the Declaration of Helsinki 2013. Ethical approval was obtained from the Mbarara University of Science and Technology Research Ethics Committee (MUST-REC, No. MUST-2021-293), and clearance to access archives for the study was granted by the Uganda National Council of Science and Technology (UNCST). Administrative clearance was obtained from the Resident District Commissioner's Office, the Mbarara City Health Officer, and the respective facility in charge. All participants provided written informed consent.

### 2.8. Positionality statement

The team was led by highly experienced and trained researchers, who are alive, work in Mbarara City, and have enough experience in HIV research. Like all qualitative studies, there might be bias in data collection and translation that may affect the findings of the study.

## 3. Results

### 3.1. Sociodemographic characteristics of the study participants

This section brings out the characteristics of the study participants. Details are provided in Table 1. Results are further presented below with narratives.

**Table 1:** Demographic characteristics of female sex workers.

Variable	Category	FSWs (*n*)	Health workers (*n*)
Age	21–30	20	5
	31–40	8	9
	41–50	2	7
Level of education	Primary	7	0
	Secondary	17	0
	Tertiary	6	21
Marital status	Single	12	4
	Married	3	15
	Separated	11	0
	Widowed	4	2
Duration in HIV care	Less than 1 year	15	0
	2–5 years	5	9
	Above 5 years	10	12

FSWs, female sex workers.

### 3.2. Healthcare workers

As seen in Table 1, most of the HCWs (9) were aged between 30 and 40 years. All 21 HCWs had attained tertiary education, and most of them (15) were married. Twelve of the HCWs have been working in HIV care for more than 5 years.

### 3.3. Female sex workers

As seen in Table 1, out of 30 FSWs, 20 were aged between 21 and 30 years. Seventeen of the FSWs attained secondary education, and those enrolled in HIV care were aged between 21 and 30 years. Eleven FSWs were single, and only three were married. Fifteen FSWs had spent less than a year in HIV care.

### 3.4. Facilitators of enrollment and retention of FSWs in HIV care

Three themes emerged as facilitators toward enrollment and retention of FSWs in HIV care to include (1) good health living, (2) receptive HCWs and availability of health services, and (3) community outreach services and peer support (Table 2).

**Table 2:** Facilitators of enrollment and retention of FSWs in HIV care.

Statements	Keywords	Subtheme	Theme
• Keep in good health and live longer • Avoid developing signs of AIDS	Good health	Good health	Good health living
• Love to keep business • Love for family • Need to produce healthy babies and taking care of them	Love for family	Love for family	
• Being sick and my child • Continuous demand for unprotected sex • Death of a partner	Continuous sickness, HIV-positive results, pregnancy, death of a partner	Fear of AIDS and other complications	
• Availability of trained HCWs • Good reception • Good reception at health centers • Care by healthcare workers	Trained HCWs Trained expert clients/peer support Reception and care	Trained HCWs Expert clients Receptiveness	Receptive HCWs and availability of HIV services
• Availability of ART • Free counseling and testing services	Counseling, ART	Counseling, access to ART	
• Follow-up and tracing lost clients • HCWs found us in business centers • Convenience of test and treatment option	Follow-up in communities, access to testing and treatment services, community outreach, assisted partner notification	Community outreach, testing and treatment, follow-up, contact tracing	Peer support and community outreaches
• Peer support • Availability of counseling services • Follow-up by HCWs	Involvement of peer helpers Support from expert clients	Peer helpers	

FSWs, female sex workers; HCWs, healthcare workers.

It consists of statements, subthemes, and themes.

#### 3.4.1. Theme 1: Good health living

This theme emerged from three subthemes; (a) good health, (b) love for their family, and (c) fear of AIDS.

##### 3.4.1.1. Good health

Across all the interviews that were conducted, the majority of FSWs revealed that the primary reasons why they enrolled and remained in HIV care were because of their wish to keep in good health and live longer, although they were HIV-positive and sexually active. Participants articulated that enrolling in HIV care would keep them healthy after receiving HIV drugs. FSWs reported that taking ART will improve their body's immunity and help them stay healthy and live longer.

*“We take those drugs because they help us to remain healthy and live long however much we remain HIV positive, and that's why most of us go to hospital” (FSW, 30 years).*

##### 3.4.1.2. Love for their family

The study findings indicated that most of the FSWs who were married with children enrolled and remained in HIV care to live healthy and accomplish their life roles, such as taking care of their children and their beloved family members. FSWs’ desire to see their children going to school encouraged them to seek HIV care services to live healthier, stronger lives and take care of their children and beloved ones.

*“I want to take care of my children and I love my self. I had a friend who was on ART, but after she stopped taking it, she later died. When I look at the orphans she left and how they are suffering … I don’t want my children to suffer like them” (FSW, 25 years).*

##### 3.4.1.3. Fear of AIDS and other complications

HIV/AIDS is characterized by a wasting syndrome and opportunistic infections (Kaposi’s sarcoma and tuberculosis). This is the most feared state of HIV among FSWs because even their customers run away from them at this stage. Having a positive HIV test status created fear among FSWs, which encouraged them to enroll and stay in care. FSWs feared that if they dropped out, they would present with the signs and symptoms of AIDS. Of the fear of HIV/AIDS, FSWs were forced to enroll in HIV care.

*“If you don’t go to the hospital and get the medicine, you become slim and develop rashes on body, even you can’t get customers because they start fearing you” (FSW, 28 years).*

*“Some of them fear to come early, but when they start losing weight, that forces them to come and test and if they test positive, we immediately initiate them on ART” (HCW, 45 years, female).*

FSWs also reported that to keep their business of sex work running, they must have a good-looking body to attract their customers, so they are forced to test, and if they find themselves positive, they initiate ART to keep looking healthy and attractive to their customers.

*“To remain in the business, you must be healthy, the customers always pick the good-looking ones” (FSW, 23 years).*

##### 3.4.1.4. Demand for unprotected sex

The FSWs verbalized a high demand for unprotected sex from their customers, and they did not test them before having intercourse; this exposed them to more risk of HIV/AIDS-related complications. However, FSWs stated that unprotected sex pays more than protected sex and most men demand it. FSWs reported that because of unprotected sex they are exposed to HIV, which also forces them to visit health facilities for testing, and most find when they are HIV-positive.

*“With a condom its 10,000 Ug shillings, without a condom we double the price, but they like unprotected sex” (FSW, 30 years).*

*“Because we always have unprotected sex, we go to test ourselves to know our HIV status” (FSW, 23 years).*

##### 3.4.1.5. Death of a partner

FSWs reported that HIV is real and it kills. They reported that some of them started testing when their partners died because of HIV. People who started on ART and later dropped out of care die, forcing the FSWs to remain committed to HIV care and taking ART on a daily basis as prescribed by the HCWs. They also stated that they know of their customers that died of HIV and that forces them to remain in HIV care so that they can live longer.

*“My husband died of HIV, that's why for me I cannot miss taking my drugs in time and on daily basis, because I know what it means when you refuse to take it” (FSW, 34 years).*

From the theme above, it can be stated that personal initiatives aimed at having good healthy living enhanced enrollment and retention in HIV care among FSWs.

#### 3.4.2. Theme 2: Trained HCWs and availability of HIV care services

This theme emerged from four subthemes detailing how the availability of services at the health facilities in addition to welcoming HCWs enabled FSWs to enroll and remain in HIV care in Mbarara City. These include (1) trained HCWs and expert clients, (2) positive attitude of HCWs, (3) counseling and testing services, and (4) availability of ART.

##### 3.4.2.1. Trained HCWs and expert clients

The Ministry of Health, with support from international organizations, has extended HIV services to every health facility and continuously provides refresher training to the HCWs and the expert clients working in the ART clinics to update them with the current guidelines. This is evidenced by the quality of services the HCWs and expert clients provide to FSWs. HCWs reported that they receive monthly training, and this helps them to provide quality care to people living with HIV, especially FSWs.

*“We receive regular trainings from Ministry of Health experts, and we always follow the World Health Guidelines while providing HIV services to FSWs” (HCW, 32 years, male).*

*“The expert clients are also trained with us, and they help us so much …”(HCW, 40 years, male).*

FSWs reported that they enroll and remain in HIV care because they know they are in the safe hands of trained HCWs. FSW also reported that each time they visit the primary health clinic/facility, HCWs provide HIV care services (HIV counseling, testing, and provision of ART). If there is evidence of poor adherence to ART or drug resistance, patients are switched to other regimen options.

*“We trust our HCWs and that keeps us going there when there is a problem or in case you need to ask them some questions” (FSW, 30 years).*

FSWs reported that they feel relieved when they find that some of them are working at the health facilities as expert clients/peer supporters, and that gives them hope in what they are doing. Furthermore, the HCWs also reported that the FSWs share their problems freely with the expert clients than they share with them.

*“When you are talking to someone who is like you, it is much easier because we share the same challenges … the expert clients help us a lot” (FSW, 25 years).*

*“They are free to share with expert clients, and that helps us to know what they do not tell us” (HCW, 35 years, female).*

##### 3.4.2.2. Positive attitude of HCWs

FSWs reported that the positive attitude of HCWs has helped them to enroll and remain in HIV care. FSWs mentioned that the HCWs have a good and receptive attitude at the health facilities. They are willing to help at any time you go there. FSWs observed that the HCWs are willing to listen to their problems and provide solutions.

*“The HCWs are receptive, and they are willing to help us, even when you call on a phone, they help…” (FSW, 35 years).*

*“Our work is to provide care for all, but we give closer attention to the FSWs because they face a lot of problems in their worker” (HCW, 40 years, female).*

##### 3.4.2.3. Counseling and testing services

The FSWs reported that the HCWs provide them with the best counseling and testing services. The FSWs reported that before anything is done, they first counsel you to get tested, and if you test positive, they initiate you on ART when you are ready. The counseling and testing services relieve stress and prepare the people living with HIV for the next steps of management and HIV care.

*“They counsel you and you feel relieved and ready to start taking ART” (FSW 30 years).*

*“We provide them with counseling services to prepare them before testing, and those who test positive, we counsel them to know how to take their ART and its importance” (HCW, 50 years, male).*

##### 3.4.2.4. Availability of ART

Participants reported that they remained in care because of the availability of ART and other services. Participants stated that every time they visit health facilities, they find ART available at the health facilities, which kept them enrolled in HIV care. HCWs also mentioned that ART drugs are always available, ensuring that individuals living with HIV receive ART every time they visit primary health facilities.

*“Every time you go to the health facility, you find drugs there, that keeps us going there” (FSW, 21 years).*

##### 3.4.2.5. Availability of other services

FSWs stated that in addition to HIV services, they also get other services at health facilities, which is why they keep going. Some of the services include treatment for other sexual infections such as gonorrhea and syphilis as well as for common diseases like malaria and typhoid. Those who are pregnant also access antenatal services. Therefore, keeping health centers equipped with HCWs and enough drugs motivated and attracted FSWs living with HIV into healthcare to improve their wellbeing.

*“When go to the health facility to pick drugs for HIV you also get other services like antenatal, if the nurses see that you have other infections, they also treat them” (FSW, 27 years).*

#### 3.4.3. Theme 3: community outreach services and peer support

##### 3.4.3.1. Female sex workers

In the HIV care system in Uganda, expert clients are trained to provide HIV care alongside HCWs to reduce the workload. The expert clients help provide counseling services and give health talks to people living with HIV. Peer support was given by fellow FSWs at health centers who worked alongside HCWs.

*“The expert clients talk exactly what they went through and they are the challenges that we go through, so we listen to what advise us to do” (FSW, 24 years).*

The expert clients do not stop at providing counseling services at the health facilities; they also follow up with lost clients in their communities and try to bring them back to the health facilities; for those who remain hesitant, the expert clients pick their drugs from the health facilities and deliver to their homes. They have played a significant role in the retention of FSWs in HIV care.

*“Expert clients help us to track their friends who drop off care by following them up to their homes and take for them drugs” (HCW, 36 years).*

Most FSWs indicated that they were recruited into HIV care through outreach services by HCWs to areas where they operate their business and in their clusters. This was done either through testing and treatment method or through contact tracing and counseling by expert clients. The community outreach services have helped to enroll many FSWs in HIV care from their communities. Community outreach services were effective in testing, enrolling, and tracing FSWs, especially those who were unknown HIV-positive initially.

*“They found me at my workplace in the bar, that where they tested me from, when I was found positive, they counselled me and I initiated on ART” (FSW, 21 years).*

### 3.5. Barriers to enrollment and retention of FSWs in HIV care

The enrollment and retention of FSWs in HIV care has faced a number of barriers. The barriers have been summarized into four major themes: (1) stigma, community discrimination, and briefs; (2) social obstacles; (3) adverse effects of ART; and (4) inadequate services at the health facilities.

#### 3.5.1. Theme 1: Stigma, community discrimination, and briefs

This theme emerged from two subthemes including fear of testing and enrolling in HIV care and fear of disclosing their status (Table 3).

**Table 3:** Barriers to enrollment and retention of FSWs in HIV care.

Statements (barriers)	Keywords	Subtheme	Theme
• Fear of testing for HIV and enrolling in care	Fear of enrolling	Fear of testing and enrolling in HIV care	Self-stigma, community discrimination, and beliefs
• Sex workers lose hope in life • Self-blame • Fear of death	Hopelessness Blame	Self-stigma	
• Family neglect after discovering their HIV status, Loss of hope in themselves, and some hate taking drugs daily • Some girls fear going for testing because of fear of being seen by others taking drugs • Fear of customers discovering their status	Discrimination Family neglect Fear of disclosing their HIV status	Discrimination Family neglect Fear of disclosing	
• Too much alcohol and drug abuse makes sex workers forget to take their drugs consistently • Lack of food, and yet drugs are strong to take while hungry • Distance from workplaces • Delay in going back home • Frequent movement when following clients	Alcoholism Forgetfulness Substance abuse Lack of food Tight schedule Lack of permanent places of work	Substance abuse Inadequate food Temporary nature of their work	Social obstacles
• I would have bad visions and would shiver at night while doing sexual activities	Dizziness, loss of work	Side effects	Adverse effects of ARVs
• Long queues and delays at the health facilities • Segregation in the community	Lines are always long at the facility	Long waiting hours	Inadequate services at the health facilities
• Shortage of supplemented drugs in hospitals for other infections like syphilis and gonorrhea	Lack of STI drugs	Inadequate supplies and drugs	
• Some HCWs are rude • Limited time at the facilities • Inadequate counseling services	Do not usually give time to patients	Poor attitude of health workers	

FSWs, female sex workers.

##### 3.5.1.1. Fear of testing and enrolling in HIV care

FSWs did not hesitate to report fear of having positive HIV test results as one of the major barriers to HIV testing and thus enrollment and retention into care. They mentioned that they fear testing, and most of them remain ignorant about their HIV status. They also reported that enrolling in HIV care is a challenge because they are seen by everyone when they go to pick medication from the health facilities, which is why most of them drop out of care.

*“Majority of the young girls fear to be tested and those that test positive fear to come and pick their drugs” (HCW, 40 years, male).*

*“Yes, it is true, we fear going to the HIV clinic to pick drugs because everybody there know that your also HIV positive” (FSW, 28 years).*

FSWs reported that having a positive HIV test means the end of life, so they do not find value in enrolling in HIV care and taking antiretroviral (ARVs). They instead blame themselves for contracting the infection from someone they do not know and also choose to spread the infection by having unprotected intercourse with other people since they also got it in the same way. The feeling of being close to death stops most FSWs from enrolling in HIV care.

*“I refused to start on the medication because I knew I was going to die soon” (FSW, 24 years).*

*“Some of them come when they have lost hope, but when we counsel and initiate them on ART, they regain their health and they are still alive” (HCW, 40 years, female).*

##### 3.5.1.2. Fear of disclosing the HIV status

Most FSWs fear disclosing their HIV status to their friends because they do not want to be recognized as HIV-positive in their communities. Even married sex workers do not disclose to their family members due to the fear of being blamed.

*“You cannot tell your secrets to everyone because when you tell them they start blaming you” (FSW, 32 years).*

#### 3.5.2. Theme 2: Social obstacles

Social obstacles refer to all social factors that act as barriers to the enrollment and retention of FSWs in HIV care. This includes the use and abuse of substances (alcohol and other drugs), inadequate food, long working hours, and the temporary nature of their work.

##### 3.5.2.1. Alcoholism and substance abuse

Most FSWs work in places of entertainment (bars and clubs), and their use of toxic substances and alcohol is high. Unfortunately, this makes them forget to take their ART as prescribed by the HCWs. They stop returning to health facilities for refills and drop out of HIV care.

*“Most of our clients take alcohol and when they are drunk, they can’t take their drugs, they even forget to come back for the refills” (HCW, 50 years, female).*

##### 3.5.2.2. Inadequate food

It is important for individuals living with HIV to have adequate and high-quality food. Adequate and high-quality food provides the required nutrients that the body needs to keep the immune system stronger and helps manage HIV-related symptoms and complications.

*“To take those drugs requires eating adequate food, and most of us do not have it, that's why some of us stop taking them” (FSW, 28 years).*

##### 3.5.2.3. Long working hours and temporary nature of their work

Most of the FSWs reported the challenging nature of their working condition, whereby they have long working hours from evening up to late in the morning. Most of them sleep during the day, and they do not find time to visit the health facilities during the day.

*“We work the whole night and sleep during the day, so we do not have time to visit the hospitals” (FSW, 25 years).*

Furthermore, FSWs do not have stable working places; they keep moving to different bars and nightclubs depending on where most clients are; thus, they keep changing points of ART refilling. It is sometimes hard for the HCWs and expert clients to trace and follow them up.

*“FSWs keep changing from one point to another, thus it becomes hard to retain them at one health facility” (HCW, 28 years, male).*

#### 3.5.3. Theme 3: ART-related side effects and adverse events

Initiating ART comes with a number of side effects. Some people gain weight, while others lose weight. Others reported that they always get nightmares, which forces them to stop taking ART.

*“Some say that when they swallow the drugs, they become dizzy, so they sacrifice taking ARVs to keep their sex job” (HCW 27 years).*

*“We know of the side effects of ART, but those stop with time” (HCW, 45 years, female).*

*“I was dreaming people burying me, but it later stopped, I would feel scared, but later I stopped dreaming and am now stable and healthy on ART” (FSW, 32 years).*

#### 3.5.4. Theme 4: Inadequate services at the health facilities

This theme emerged from three subthemes, including inadequate supplies and drugs, long waiting hours, and poor attitude of some HCWs.

##### 3.5.4.1. Inadequate supplies and drugs

Due to the low socioeconomic status, FSWs reported difficulties purchasing non-ART drugs and medical kits in case health facilities were out of stock. FSWs reported finding it difficult to buy or purchase medications from their own pockets and are discouraged from returning to health facilities that do not have adequate drug supply or provide all services.

*“When you go to the hospital twice and there are no drugs you feel discouraged to go back there” (FSW, 34 years).*

##### 3.5.4.2. Long queues and waiting hours

FSWs reported that they wait for many hours and in long queues in health facilities. They highlighted that it is discouraging to spend long hours in long queues.

*“Long ques and delays at the health facilities discourage us” (FSW, 20 years).*

*“We receive very many patients here and we have to work on them all, so sometimes they delay receiving care, but that is a general challenge” (HCW, 40 years, female).*

##### 3.5.4.3. Negative attitude of some health workers

FSWs reported that sometimes the HCWs are rude, which discourages them from coming to health facilities, mostly if they get to know which HCWs are on duty. If it is the rude ones, they find themselves missing the appointment dates. However, the HCWs attribute this to the heavy workload, and they are already tired sometimes. They also tend to be rude when some FSWs skip their appointments for a long time. Such clients fear being reprimanded and subsequently will miss their appointment dates.

*“Some HCWs are very rude, to us, and that discourages us from coming back to pick the drugs” (FSW 34 years).*

## 4. Discussion

Enrollment and retention in care are essential in HIV care continuum and necessary for the successful management of HIV infection. This study adds to the existing literature by exploring the enrollment and retention of FSWs in HIV care.

### 4.1. Facilitators of enrollment and retention of FSWs in HIV care

The need for every HIV-positive female sex worker to have good health and long life encourages them to enroll and remain in HIV care. Other studies reported that the desire to remain healthy, get married, and have children, in addition to the knowledge they had of the benefits of early treatment (19), kept FSWs enrolled in HIV care. This means that to remain healthy, FSWs must continue taking ART as prescribed. FSWs living with HIV know that taking ART is the only way to boost their immune system, stay healthy, and live longer. HCWs should continue health-educating the FSWs on the importance of ART for their lives and why they should remain in HIV care once enrolled.

Refresher training to the HCWs and expert clients working in the ART clinics to update them with the current guidelines improves the quality of services they provide to FSWs and promotes retention in HIV care. Results from other studies show that trained expert clients provide excellent health education, counseling, and outreach services, and their involvement is acceptable to most FSWs. Expert clients established strong personal relationships with other FSWs, thus improving enrollment and retention of HIV-positive FSWs in HIV care (20). Continuous training should be implemented, and expert clients should be fully integrated into HIV care.

FSWs feel relieved when they find expert clients at health facilities. The HCWs also reported that the FSWs are free to share their problems with the expert clients than they would wish to share with health workers. It is reported in other studies that peer support with routine medical care is superior to routine clinic follow-up in improving outcomes for people living with HIV. The approach was also recommended as feasible and effective for linking and retaining HIV-positive sex workers to HIV care, which can help HCWs shoulder existing services (21, 22).

The positive attitudes of HCWs were found to have greatly influenced FSWs to enroll and remain in HIV care. The good and receptive attitude the FSWs find at the health facilities and the willingness to help and listen to their problems and provide solutions to their problems attracted HCWs to remain in HIV care. Similarly, attitudes of HCWs are positively associated with retention of FSWs on ART, and retention was 20% higher among the sex workers that perceived the HCWs’ attitude to be good compared to those that perceived it to be fair (23). Measures to improve the attitudes of HCWs will greatly improve the enrollment and retention of HIV-positive FSWs in HIV care.

HIV counseling is key in the fight against stigma and in improving retention and enrollment in HIV care. With the best counseling services, FSWs reported willingness to start ART (21, 23). Therefore, the HCWs, in addition to clinical knowledge, should be equipped with knowledge and skills on how to provide HIV care services to FSWs living with HIV.

Provision of other healthcare services in addition to HIV, such as screening, testing, and treatment of sexually transmitted infections (STI), malaria, and typhoid, has motivated and attracted HIV-positive FSWs to enroll and remain in healthcare services, thus improving their wellbeing. Studies conducted in sub-Saharan Africa reported that the integration of HIV care services into non-HIV services (maternal, child healthcare, reproductive health services) had improved the enrollment and retention of FSWs in HIV care (24, 25). Therefore, integration of HIV care services into other routine health services is most likely to improve patient outcomes and overall efficiency of healthcare systems.

In Uganda, expert clients are trained to provide certain HIV care services such as counseling and health education, and this is done as part of task shifting and trying to reduce the workload for HCWs. Various studies have reported that FSWs living with HIV feel safe when assisted by other FSWs (peer experts) in the healthcare facility. In addition, peer experts are beneficial in helping fellow FSWs join clinic queues to receive ART medication faster, helping pick and deliver ART medication for those who cannot attend healthcare clinics, and providing psychosocial support (26). Peer supporters were very crucial in the enrollment of FSWs with respect to HIV healthcare and also in providing them with psychosocial support.

Our findings showed that some FSWs are recruited into HIV care through outreach services by HCWs to areas where they operate their business and in their clusters. The community outreach services have enabled us to enroll many FSWs in HIV care from their communities. It is reported that community initiatives such as outreach services by HCWs facilitated enrollment and retention of FSWs in HIV care. It is during such visits that FSWs who had never tested got to know their HIV status, while those who knew their status but feared to enroll were recruited (10). However, the shortage of HIV diagnostics and supplies hindered community-based HIV testing and delivery services and kept most FSWs away from care (27). Whereas community outreach services provide effective models for recruiting FSWs into HIV care, more strategies are required to link them to mainstream health facilities where they may receive consistent HIV care services.

### 4.2. Barriers to enrollment and retention of FSWs

HIV is associated with stigma and segregation from family and community members; however, it is worse when associated with sex work, which is a condemned and cursed business in Uganda, and that is why most sex workers do not enroll in HIV care. Stigma is a process by which an individual is labeled based on characteristics that may not adhere to socially accepted norms, resulting in reduced wellbeing and opportunities for that individual. Sex workers may experience stigma due to their engagement in sex work, HIV status, or other identities and behaviors (drug use, gender identity, sexual orientation, migration status) and the intersections of these characteristics. HIV-related stigma could be based on the actual or perceived status of living with HIV. This can lead to fear or avoidance of health services (28).

Fear of having a positive HIV test result has remained one of the major barriers to HIV testing and thus enrollment and retention in care. It has been mentioned that the fear of testing stopped most FSWs from enrolling and retaining in HIV care. Other studies reported that the gap between testing and enrollment among FSWs due to fear of testing and being seen by everyone when they are going to pick medication from the health facilities might reduce their chances to enroll in HIV care, and this could be the reason why most of them drop out of care (7). HCWs together with other stakeholders should strive to make the FSWs aware of the importance of early testing and treatment of HIV.

Discrimination, self-stigma, and segregation were found to be the major causes of poor health-seeking behaviors among the FSWs. Most FSWs were hesitant to seek HIV care due to the fear of being known or identified as HIV-positive. Fear of being known as HIV-positive led to clinic avoidance behaviors among HIV-infected FSWs. Home-based care interventions such as counseling were moved away from home to prevent neighbors and others from knowing about their participation in the intervention and, by inference, their HIV serostatus (29, 30). It was established that the main barriers to enrollment in HIV care among FSWs were perceived stigma, fear of being seen at outreach HIV clinics, fear and myths about antiretroviral therapy, lack of time to attend a clinic, and financial constraints (31). Extending HIV care services to communities can mitigate fear and stigma and may improve the retention of FSWs in care. Family members and close friends also tended to discriminate against them when they discovered that they were HIV-positive, and this affected their enrollment and retention in HIV care. Facilitated serostatus disclosure in the presence of trained health workers would help to solve major challenges associated with HIV disclosure.

Most FSWs work in bars and nightclubs where they got exposed to toxic substances and alcohol to keep them energetic and to work throughout the night. These substances however make them oversleep and forget to take their ART. This has further made the situation worse because the use of substances interferes with consistency in enrollment and retention in HIV care. Other studies reported that alcohol and drug abuse were among the barriers to the retention of FSWs in HIV care (5). Therefore, continuous health education on the dangerous effects of alcohol and other substances while on ART could reduce their use by HIV-positive FSWs.

Substance use, particularly alcohol use, has been reported as a barrier to HIV care among FSWs. Currently, there are few interventions related to HIV care that focus on reducing substance use among FSWs living with HIV (32). Substance use can impair cognitive functions, which may adversely affect health-seeking behaviors such as enrolling and retaining in HIV care (9).

In southern Africa, food insecurity has been linked to an increased risk of contracting HIV and difficulty remaining in HIV care for FSWs living with HIV. Food insecurity is defined as the state of having uncertain or limited access to sufficient and nutritious food (1). Food insecurity has been linked to high-risk sexual practices among women. This forces women to look for ways to provide food for their families that involves high-risk sexual practices that include sex work, transactional sex, and decreased condom use (1–4). It was further reported that FSWs in sub-Saharan Africa have suboptimal HIV care and treatment engagement outcomes as a result of poor nutrition, food insecurity, and substance use (9), which were commonly reported and associated with poor ART initiation and retention in care. While engagement in sex work can be income-generating, FSWs may continue to struggle with food insecurity (9). Measures to improve food security would improve retention in care of FSWs living with HIV and minimize the risks of sex work among young females.

The main challenges faced by FSWs include poor working conditions, health risks, violence, discrimination, poor access to substantial healthcare services, legal assistance, and social services. Due to their working hours, they often struggle to find time to visit healthcare facilities as they prefer to rest or sleep during the day to be productive in the evenings (33). Unfortunately, the nature of their work and its environment bring stress with cases of sexual assault and harassment noted in some instances (33). These issues have been reported as barriers for them to access HIV care services.

Due to their low economic status, FSWs find it hard to buy drugs and attain other healthcare services, such as pregnancy test kits and family planning, that are not offered in public health facilities when they are out of stock; high cost of other health services (health system) hinders retention of FSWs in HIV care (19).

Health facilities should be equipped with the required supplies so that they can offer integrated services to FSWs at any point of their visit to the health facilities.

FSWs spend long waiting hours at the facilities, which discourages them from further coming and hinders their retention; this is due to the unfavorable working conditions. Studies demonstrated that HCWs’ shortage and high workload led to poor care provisions and long wait times for individuals living with HIV, contributing to suboptimal retention (30). Increasing the number of HCWs and expert clients could decrease the workload and the long waiting hours at the health facilities.

In some cases, HCWs are reported to mistreat FSWs and judge them for the kind of work they do, creating reluctance in routine facility visits. HCWs have attributed their negative attitudes toward FSWs to heavy workloads and abnormal working hours causing fatigue and burn out, which compromises the quality of work offered to FSWs (16, 19, 31).

Various studies have reported that the shortage of clinicians and lay health workers in healthcare facilities, accompanied by high workload, is linked to poor provision of services, poor quality of care, and long waiting hours for individuals living with HIV, leading to poor retention in care (30).

## 5. Conclusion

FSWs are challenged by the unsupportive environments and communities where they live and work, which hinders their enrollment and retention in HIV. It is important to integrate sex work-related services into other services within health facilities. Food security among FSWs such as developing comprehensive food security interventions for FSWs should be be integrated with HIV programs. Creating awareness of the utilization of HIV care services and extending such services to the communities could positively impact the lives of FSWs.

## Data Availability

The raw data supporting the conclusions of this article will be made available by the authors, without undue reservation.

## 6. Limitations of the study

Being a qualitative study design, some of the participants might not have provided relevant information during interviews; however, much data collection was done at the convenience of the participant. This might have left a lot of information not captured in the study.
